# WU Polyomavirus in Fecal Specimens of Children with Acute Gastroenteritis, China

**DOI:** 10.3201/eid1501.080693

**Published:** 2009-01

**Authors:** Lili Ren, Richard Gonzalez, Xiwei Xu, Jianguo Li, Jing Zhang, Guy Vernet, Gláucia Paranhos-Baccalà, Qi Jin, Jianwei Wang

**Affiliations:** State Key Laboratory for Molecular Virology and Genetic Engineering, Beijing, People’s Republic of China (L. Ren, Q. Jin, J. Wang); Institute of Pathogen Biology, Beijing (L. Ren, R. Gonzalez, J. Li, J. Zhang, Q. Jin, J. Wang); Fondation Mérieux, Lyon, France (R. Gonzalez, G. Vernet, G. Paranhos-Baccalà); Beijing Children’s Hospital–Capital University of Medical Sciences, Beijing (X. Xu)

**Keywords:** WU polyomavirus, feces, fecal specimens, gastroenteritis, letter

**To the Editor:** WU polyomavirus (WUPyV) is a recently described PyV found in patients with acute respiratory tract infections (*1*). The role of the virus in disease pathogenesis remains unclear. The ability to detect it in clinical specimens would help in the determination of its replication sites and its routes of transmission and dissemination. WUPyV has been found in specimens from the respiratory tract only (*1*).

Previous studies of other PyVs, including BK virus, JC virus, and the newly identified KIPyV, demonstrated their presence in fecal specimens (*2*,*3*), which suggests their potential for transmission through the gastrointestinal (GI) tract (*2*). Because some children (6.8%–27.7%) who had WUPyV results in previous studies (*1*,*4*,*5*) displayed respiratory and GI clinical signs, we speculated that WUPyV might also be transmitted through the GI tract.

In this study, we tested for the presence of WUPyV in children with acute gastroenteritis. A total of 377 fecal specimens were collected from children with acute nonbacterial gastroenteritis at the Outpatient Clinic Department of the Beijing Children’s Hospital from March 2006 through November 2007. Patients with nonbacterial gastroenteritis were defined as 1) those who had acute, watery, but not bloody, diarrhea, accompanied by other clinical signs and symptoms such as fever, abdominal cramps, nausea, vomiting, and headache; and 2) those who had negative test results for any known bacteria that might cause gastroenteritis, such as *Salmonella* spp*., Shigella* spp.*, Staphylococcus* spp*., Campylobacter jejuni, Clostridium* spp.*, Escherichia coli,* and *Yersinia* spp*.*

All patients, whose ages ranged from 1 month to 13 years (mean age 11.7 months, median age 9 months), did not exhibit apparent clinical respiratory signs. Fecal specimens from patients were diluted in phosphate-buffered saline (pH 7.2) by using a 10% wt/vol ratio and were cleared of cell debris by centrifugation (2,500 × *g*, 5 min). Virus nucleic acids were extracted by using the NucliSens miniMAG and isolation reagents according to the manufacturer’s instructions (bioMérieux, Marcy l’Etoile, France). Samples were subsequently screened for group A rotavirus (RVA) by using the rotavirus ELISA diagnostic kit (Lanzhou Institute for Biologic Products, Lanzhou, People’s Republic of China). In addition, samples were screened for enteric adenovirus, astrovirus, norovirus, and human bocavirus by PCR (*6*,*7*). WUPyV DNA was detected by PCR with the primer pair AG0048 and AG0049, which generated a 250-bp amplicon as described previously (*1*). Positive PCR amplicons were then verified by sequencing. Confirmed sequencing results demonstrated WUPyV DNA in 2 (0.5 %) of 377 fecal specimens. These 2 positive samples were obtained from 2 patients, ages 6 months and 2 years, who experienced acute diarrhea but had no respiratory or other clinical signs and symptoms. RVA was also detected in both samples.

Nucleotide sequences of WUPyV obtained from this study were submitted to the GenBank database (accession nos. EU684312 and EU684313). To investigate whether these nucleotide sequences had any unique features, we analyzed the 2 WUPyV isolates to determine the extent of homology between these genes and those documented in the GenBank database by using MEGA software version 4.0 (www.megasoftware.net) and the neighbor-joining method. The nucleotide sequences of the VP2 gene from the WUPyV strains found in this study showed 99%–100% homology with the strains described previously for WUPyV (GenBank accession nos. EU754877, EU754878, EF444557, EF444562, EF444593, EF655819, EU296475, EU395815, EU678910, EU693905, EU358752).

Our observations indicate that a candidate respiratory pathogen, WUPyV, can also be detected in specimens from the GI tract. In addition, the codetection of human RVA, a major cause of viral gastroenteritis in children, in both WUPyV-positive specimens underscores the need for further investigations to clarify the precise role of WUPyV in the pathogenesis of acute gastroenteritis. The reason for the presence of WUPyV in the GI tract is unclear. Our findings were unlikely to have been caused by cross-contamination because samples were prepared and analyzed in 2 laboratories independently, and strict controls were used during the process of nucleic acid extraction and PCR analysis to monitor contamination.

WUPyV may act as an opportunistic pathogen in the GI tract, colonize the GI tract without causing any disease, or be a part of the endogenous viral flora that are reactivated by other viral infections (*1*). However, although positive samples were obtained from patients who had acute gastroenteritis without any apparent clinical respiratory symptoms, we cannot exclude the possibility that the detection of WUPyV in fecal specimens might result from its transient presence in patients who have swallowed virus-containing sputum or nasal secretions. It is also possible that WUPyV persists in the respiratory tract without inducing symptoms (*8**,**9*). Thus, the study of asymptomatic control groups of patients with diarrhea would be of particular interest because these patients may provide critical insight into the pathogenesis of WUPyV.
